# An Approximate Method for Calculating Elastic–Plastic Stress and Strain on Notched Specimens

**DOI:** 10.3390/ma15041432

**Published:** 2022-02-15

**Authors:** Maxim Lutovinov, Radim Halama, Jan Papuga, Michal Bartošák, Jiří Kuželka, Milan Růžička

**Affiliations:** 1 Faculty of Mechanical Engineering, Czech Technical University in Prague, Technická 4, 16607 Prague, Czech Republic; maxim.lutovinov@fs.cvut.cz (M.L.); jan.papuga@fs.cvut.cz (J.P.); michal.bartosak@fs.cvut.cz (M.B.); jiri.kuzelka@fs.cvut.cz (J.K.); milan.ruzicka@fs.cvut.cz (M.R.); 2 Faculty of Mechanical Engineering, VSB-Technical University of Ostrava, 17.listopadu 2172/15, 708 00 Ostrava, Czech Republic

**Keywords:** plasticity, multiaxial loading, pseudostress, stress–strain estimation, 2124-T851

## Abstract

The paper deals with an approximate method for calculating elastic–plastic stresses and strains on the surface of notched samples. The method is based on the Abdel–Karim–Ohno cyclic plasticity model. The plane stress condition is considered within the evaluation. The output of the approximation on several multiaxial axial–torsion load paths is compared to our own experimental results. Experiments were carried out on samples of two notch types manufactured from the 2124-T851 aluminum alloy. Strain distribution in the notch area was measured by digital image correlation. The comparison between computational solution and measured response shows that the new method allows for obtaining reasonably good approximation, even for relatively complicated multiaxial load cases.

## 1. Introduction

Most initially isotropic engineering materials exhibit elastic–plastic behavior. However, the finite-element calculation of plasticity is time-consuming and requires more input data. Engineers dealing with fatigue life estimation, therefore, often use approaches that consider only elastic behavior to assess the durability of structures. Such a simplification is possible in the domain of high-cycle fatigue, but it is unacceptable if the low-cycle regime is evaluated, where the scale of plasticity is much more substantial. In order to assess the elastic–plastic stress state, approximate methods that take elastic–plastic material behavior into consideration might be a good alternative to time–consuming finite-element elastic–plastic analyses whenever a longer load history should be analyzed.

Many methods have already been suggested for the estimation of elastic–plastic stresses and strains. The first group of methods contains those intended for monotonic loading only [1,2,3,4,5]. The methods do not take cyclic hardening or cyclic softening into account, and they do not describe the movement of the yield surface. Therefore, this group of methods is not suitable for cyclic loading. The investigation of these methods can be found in [6].

The second group of methods [7,8,9,10,11,12,13,14,15,16] deals with cycling loading and incorporates plasticity models to describe cyclic hardening or softening and yield surface movement. Unlike finite-element analyses (FEA), approximate methods do not deal with elastic–plastic stiffness matrices to obtain a solution. Instead, they use an elastic solution that they convert into an elastic–plastic solution by using a relation either between pseudomaterial and real material or between linear–elastic and elastic–plastic strain energies.

Barkey introduced one of the first elastic–plastic stress–strain approximation methods for cyclic loading [7]. Nominal stresses and plastic strains were related in his work in order to retrieve the approximation. The kinematic work-hardening model of Mróz [17] was used to describe elastic–plastic behavior. Barkey reported good correlation between estimates with the experiments and FEA.

Two approaches, pseudonotch stress and pseudonotch strain, were presented in [8]. Both approaches incorporated the Mróz model of plasticity. The essence of the methods is in relating entities from a purely elastic solution to real elastic–plastic ones, e.g., the equivalent pseudostress to real plastic equivalent strain, creating a pseudomaterial with it. The behavior of the pseudomaterial is simultaneously characterized by stresses from the elastic solution and by plastic strains from the real elastic–plastic material response.

In conjunction with Koettgen’s model, Langlais [9] used the infinite surface hardening rule, which is a modification of Mróz’s model presented by Chu [18]. Instead of the flow rule, Langlais used Drucker’s equation, which related plastic strain rate and generalized plastic modulus. Langlais reported that the same level of precision was achieved as in [7,8].

Firat [10] used a pseudostress method similar to the method presented by Koettgen. The approach was combined with the rate-independent plasticity model by Chaboche [19]. The author reported a high accuracy of the predicted values.

Ince et al. [11] combined the Prandtl–Reuss flow rule [20,21], an assumption about the equivalence of increments of the total distortional strain energy density, and the Garud multisurface plasticity model [22]. The authors reported nonconservative estimates, as strain ranges were predicted to be 4–15% smaller than the experimental results that had been used for comparison. Regarding energy approaches, an overview of the equivalent strain energy density (ESED) approach development by Glinka and coworkers is described in [23].

Ye et al. [12] proposed a new unified expression based on the thermodynamic analysis of cyclic plastic deformation. The authors used this solution with the material constitutive model proposed by Jiang and Sehitoglu [24] to estimate notch stresses and strains. The mean relative errors were reported to be −3.5% for the axial strain component, and −3.9% for the shear strain component. The authors indicated that the unified expression developed in the paper had the range of applicability limited to chosen geometries and loading conditions, and that further verifications of the proposed approximate method were needed.

One of the recent works on the topic of pseudocurve approaches was conducted by Li et al. [13]. They combined the pseudostrain method with the Jiang–Sehitoglu plasticity model. The authors reported reasonable results under multiaxial cyclic axial–torsion loading.

The approach in [14] used tangent moduli of pseudo and real curves to calculate the real stress history. The Garud plasticity model was used to describe the behavior of the material. Experimental notch strain data were presented for samples from the TC21 titanium alloy. To the authors’ knowledge, it is the first work in which notch stress–strain correction method estimates were validated on other material than steel.

Similarly to [14], Li et al. [15] used tangent moduli for stress estimation, but their method also took into account the influence of temperature. This was achieved by incorporating the Ramberg–Osgood equation in high-temperature form [25]. Estimates were validated against FEA.

Kraft and Vormwald [16] combined the unified expression of [12] with the Ohno–Wang plasticity model [26]. The integration algorithm used to calculate the elastic–plastic variables was described in depth.

One drawback of some of the methods mentioned above, also noted by other authors [13], is that the methods are not sufficiently described for replication by other research teams. The second drawback of the current state of such methods is that experimental verification was only carried out on a limited number of materials and test paths (Table 1).

Table 1 shows that most of the materials used for the validation of the methods were steel types, and only four sources of experimental data were used. This proves that a wider experimental investigation is necessary to explore the range of applicability of such methods.

The present paper introduces a new combination of the pseudostress method and the Abdel–Karim–Ohno (AKO) plasticity model [27]. The article describes the pseudostress–plastic strain approach. Validation was performed on the own new experimental data measured on notched samples manufactured from the 2124-T851 aluminum alloy and on available experimental data of 1070 steel [7] and TC21 titanium alloy [14]. The AKO model has its advantages in an additional parameter that influences the ratcheting response. It can be enhanced by a memory surface introduction [28] in a future application of the pseudostress–plastic strain approach.

## 2. Materials and Methods

### 2.1. Approximate Method

**Figure 1:**
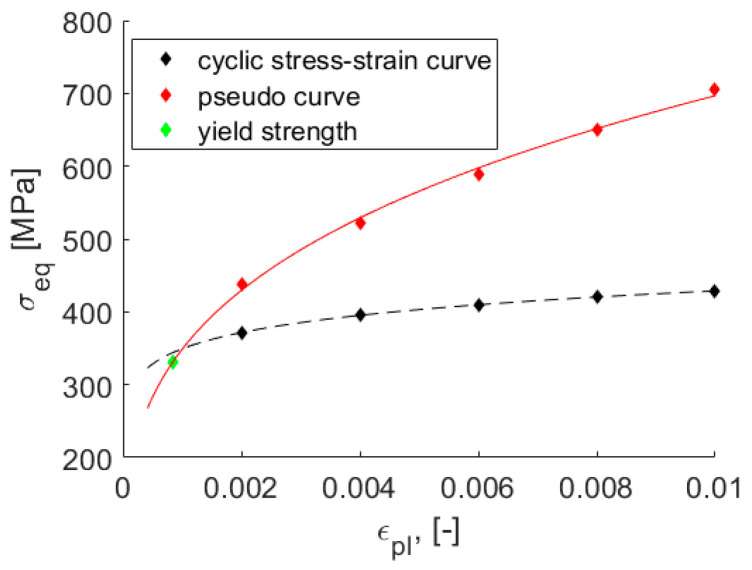
Pseudostress–real plastic strain curve and cyclic stress–strain curve; points represent discrete versions of curves based on the Hollomon parameters.

Pseudomaterial approaches are based on material behavior that couples either elastic stress with elastic–plastic strain or elastic strain with elastic–plastic stress. The behavior of the material can be represented by a pseudocurve that is analogous to the static/cyclic stress–strain curve (Figure 1). In the case of the pseudostress–real plastic strain curve, which is used in this work, the pseudomaterial experiences stresses that correspond to the stresses related to a purely elastic solution, while it plasticizes according to its real material response at the same time.

There are two types of pseudostress approaches. In the first approach, pseudostress is paired with the total strain [13]; in the second type, pseudostress is paired with the plastic strain [8,10]. The main difference between the two approaches is in the strain component that is obtained when a plasticity model is applied to pseudostress history, namely, if it is a total strain tensor or a plastic strain tensor. In the present paper, the second type of solution is used.

#### 2.1.1. Establishing Pseudostress Material Curve

A pseudocurve was established by combining elastic stress with plastic strain. The plastic strain values were the same as the plastic strain values of the real cyclic stress–strain (CSS) curve. The CSS curve could be obtained by the Ramberg–Osgood expression using Hollomon parameters (Section 2.3). Because the number of selected plastic strain values was finite, both curves were discrete.

Elastic stress was calculated on the basis of the modification of the Neuber rule:

(1) $$\sigma_{e}=\sqrt{\sigma \left(\varepsilon_{p}+\frac{\sigma }{E}\right)E},$$

where σ and $\varepsilon_{p}$ are real stress and plastic strain described by the cyclic stress–strain curve, respectively, and *E* is Young’s modulus.

When the curve was established, the parameters of plasticity model *C_i_* and *γ_i_* were calculated. The number of intervals between discrete points *i* of pseudo or real curves affected the number of backstresses used in the approximation because pairs of *C_i_* and *γ_i_* parameters were calculated here for each discrete interval of either a pseudo or real curve, and the number of pairs of *C_i_* and *γ_i_* gave the number of backstresses.

During the approximation process, curves were represented solely by the *C_i_* and *γ_i_* parameters. They are not referenced in any other way.

#### 2.1.2. Getting Real Strain and Real Stress

When parameters *C_i_* and *γ_i_* representing pseudo and real curves had been defined, the plasticity model was applied to the elastic stress history. Elastic stress history can be obtained, e.g., for a chosen loading path from an elastic FEA. Because of the way the pseudocurve was built, this step provided a real plastic stress tensor and accumulated plastic strain as its outputs. Detailed analyses of this property of pseudomaterial are presented in Section 2.1.3.

Once the plastic strain tensor had been obtained, the plasticity model was applied again, and the real stress and real total strain were estimated. However, this time, the incremental algorithm to acquire the accumulated plastic strain was not involved because it had been calculated in the previous step.

#### 2.1.3. Equivalence of Pseudo and Real Plastic Strain Tensors

The key part in calculating the real response from the pseudovariables is the equivalence of the accumulated plastic strain *dp* of the pseudocurve and of the real stress–strain curve. This was ensured by the way the pseudocurve had been established (Section 2.1.1).

Unlike the case of accumulated plastic strain, the equivalence of the pseudoplastic strain tensor and of the real plastic strain tensor was not explicitly stated in [8] or in [10], where similar approaches were used. However, it was stated in [8] that applying the plasticity model to the pseudostress history results in a real plastic strain tensor. This statement supports the claim of plastic strain tensor equivalence.

Justification can be found when analyzing the widely used relationship between accumulated plastic strain increment *dp* and plastic strain tensor increment $d\varepsilon_{p}$

(2) $$dp=\sqrt{\frac{2}{3}d\varepsilon_{p}:d\varepsilon_{p}},$$

and the flow rule:

(3) $$d\varepsilon_{p}=\frac{3}{2}dp\frac{s-a\;}{\sigma_{y}}$$

where s is the deviatoric part of the stress tensor, a is the deviatoric part of the backstress, and $\sigma_{y}$ is yield strength. *dp* and $\sigma_{y}\;$ were identical for the real curve and the pseudocurve.

Due to the intrinsic difference between the elastic and elastic–plastic material behavior of isotropic materials, the real stress was smaller than the pseudostress under the same load. Backstress α followed the stress while maintaining the radius of the yield sphere during the kinematic hardening. Because of this, it was safe to assume that tensors s−a  for the real and the pseudomaterial similarly changed; more specifically, the corresponding components of s−a  changed in a similar manner for both materials. If one component increased for the pseudomaterial, the corresponding component of the real material also increased. In other words, the increments should have at least the same sign.

The increment of plastic strain was obtained by multiplying  s−a. Therefore, the same assumptions as for s−a tensor were valid for the increment of plastic strain. However, then, if all the corresponding components of the real/pseudoplastic strain changed in a similar manner by either increasing or decreasing, they could not provide the same *dp* in Equation (2) unless they were equal. Hence, the components of pseudo and real plastic strains had to be the same.

#### 2.1.4. Approximation Method Step by Step

This section summarizes the approximation method described in Section 2.1.1 and Section 2.1.2:

1. Pseudomaterial curve is established.
2. Pseudostress history is obtained either by elastic FEA or using stress concentration factors [10].
3. The plasticity model is applied to the pseudostress history. In this step, plasticity parameters *Ci* and *γi* obtained for the pseudomaterial are used. The plastic strain tensor and the accumulated strain are calculated.
4. The plasticity model is applied to the plastic strain tensor obtained and to the accumulated strain. In this step, the plasticity parameters *Ci* and *γi* for the real material are used. Real stress and real backstress are calculated.

### 2.2. Abdel–Karim–Ohno Plasticity Model

The time-independent theory of plasticity was considered because the aluminum alloy is not sensitive to the strain rate. It was assumed that the total strain tensor was composed of elastic and plastic strain tensors $\varepsilon_{e}$**,** $\varepsilon_{p}$ according to the additive rule:

(4) $$\varepsilon =\varepsilon_{e}+\varepsilon_{p}.$$

Stresses are computed using Hooke’s law:

(5) $$\sigma =D\text{:}\varepsilon_{e}\;,$$

where D is the elastic stiffness tensor of the fourth order. The yield surface is introduced in the deviatoric space based on the von Mises condition:

(6) $$f\left(\sigma \right)=\sqrt{\frac{3}{2}\left(s-a\right)\text{:}\left(s-a\right)}\;–\;\sigma_{Y}=0,$$

where $\sigma_{Y}$ represents the radius of the yield surface (and yield strength). The flow rule and the accumulated plastic strain were defined in Equations (2) and (3). According to Chaboche [29], the backstress is constructed by superposing relevant parts of the backstresses:

(7) $$a=\sum_{i=1}^{M}a^{\left(i\right)}.$$

The kinematic hardening rule is introduced in accordance with Abdel–Karim and Ohno theory ([27], AKO), i.e., the evolution of backstress is defined by the differential equation:

(8) $$da^{\left(i\right)}=\frac{2}{3}C_{i}d\varepsilon_{p}-\mu_{i}\gamma_{i}a^{\left(i\right)}dp-\gamma_{i}H\left(f_{i}\right)\langle d\lambda_{i}\rangle a^{\left(i\right)},$$

where

(9) $$f_{i}=\frac{3}{2}a^{\left(i\right)}\text{:}a^{\left(i\right)}-{\left(\frac{C_{i}}{\gamma_{i}}\right)}^{2},$$

(10) $$d\lambda_{i}=d\varepsilon_{p}:\frac{a^{\left(i\right)}}{C_{i}/\left(\gamma_{i}\right)}\;–\;\mu_{i}dp.$$

In Equations (8)–(10), $C_{i}$, $\gamma_{i}$ are basic material parameters, $\mu_{i}$ is the ratcheting parameter, the symbol 〈x〉 represents Macaulay brackets (〈x〉=(x+|x|)/2) and $H\left(f_{i}\right)$ is the Heaviside step function. Under uniaxial loading, the model gives plastic shakedown for $\mu_{i}=0$ for all i. Then, more precisely, the multilinear model of Ohno and Wang [26] is obtained. This option is called OWI hereafter (Ohno-Wang model I, see [26]). The maximal ratcheting rate is given by $\mu_{i}=1$ for all i (Armstrong and the Frederick rule [30]), and the corresponding option is marked as CHAB (Chaboche model [19]).

The same value of the ratcheting parameter is usually set for all backstress parts, $\mu_{i}=\mu$ for all i, and its value is usually small. Therefore, the AKO model is calibrated in the same way as the OWI model [26]. The selected points of the cyclic stress–strain curve directly define the values of the basic material parameters by relations

(11) $$C_{i}=\frac{\sigma_{a\left(i\right)}-\sigma_{a\left(i-1\right)}}{\varepsilon_{ap\left(i\right)}-\varepsilon_{ap\left(i-1\right)}}-\frac{\sigma_{a\left(i+1\right)}-\sigma_{a\left(i\right)}}{\varepsilon_{ap\left(i+1\right)}-\varepsilon_{ap\left(i\right)}}\;\mathrm{for}\;i\neq M,$$

(12) $$C_{M}=\frac{\sigma_{a\left(M\right)}-\sigma_{a\left(M-1\right)}}{\varepsilon_{ap\left(M\right)}-\varepsilon_{ap\left(M-1\right)}},$$

(13) $$\gamma_{i}=\frac{1}{\varepsilon_{ap\left(i\right)}}\;\mathrm{for}\;\mathrm{all}\;i,$$

where $\sigma_{a\left(i\right)},\;\varepsilon_{ap\left(i\right)}$ are the stress amplitude and the plastic strain amplitude of the given i-th point, respectively. The resulting basic material parameters for the 2124-T851 aluminum alloy are listed in Table 2.

### 2.3. Experimental Data

**Figure 2:**
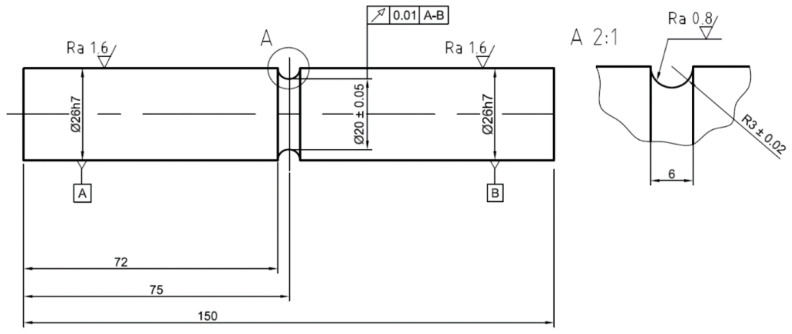
Specimen with U-notch. A and B in frames indicate surfaces based on which a datum axis for geometrical run-out tolerance is defined.

**Figure 3:**
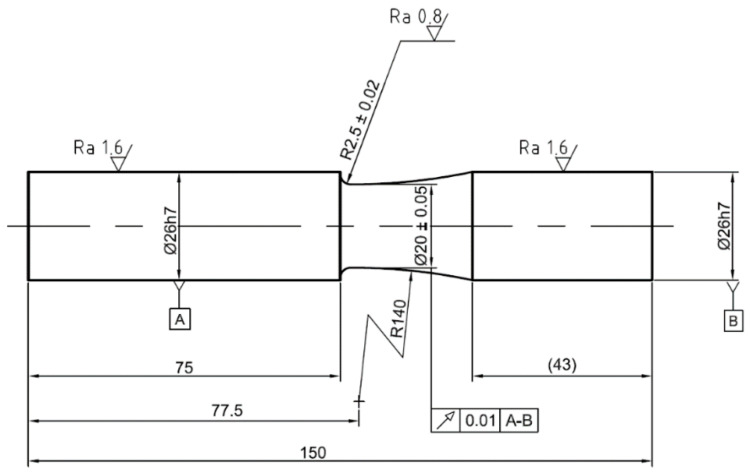
Specimen with fillet. A and B in frames indicate surfaces based on which a datum axis for geometrical run-out tolerance is defined.

To verify the approximation method, fatigue experiments were carried out on two types of notched samples (Figure 2 and Figure 3) manufactured from aluminum alloy 2124-T851. The Young’s modulus of the material was 73,100 MPa [31], Poisson number was 0.33, and Hollomon parameters for the Ramberg–Osgood curve were *K* = 646 MPa and *n* = 0.089. Cyclic yield strength $\sigma_{y}$ of 330 MPa was used for the FEA simulation and for the approximation method.

The aim of the experiments was to measure the notch tip strains, specifically the axial and shear strain components. These components are commonly used to compare estimates with experimental results [7,8,9,10,11,12,13,14,15,16].

Experiments were carried out under force and moment control. The first reason for the load-controlled experiments is the possibility of recalculating the loading forces and moments into the local elastic notch stress history using the stress concentration factors as described in [10]. However, in this work, the elastic FEA was used to obtain the local stress history. The second reason for using force control is that the strain control of notched specimens would require complex real-time notch strain measurement and processing.

**Figure 4:**
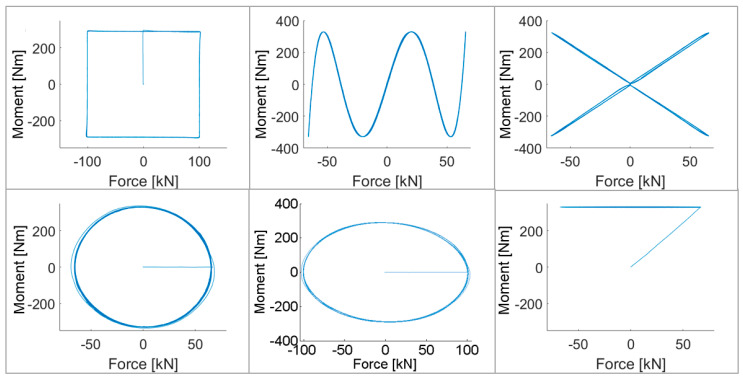
Loading paths: (**top left**) Square; (**top middle**) NV; (**top right**) X; (**bottom left** and **bottom middle**) Circle; (**bottom right**) 7.

The stress ratio of nominal axial stress to shear stress was 1 for paths Square, NV shape, X, and path 7 (see Figure 4). In the case of path Circle, results corresponding to stress ratios 1 and 1.73 are presented. For stress ratio 1, maximal force was 65.8 kN, and maximal moment was 329 Nm. For the 1.73 ratio, maximal force was 100.5 kN, and maximal moment was 290 Nm. Experiments were carried out at room temperature.

Path Square was achieved by multiaxial loading by trapezoidal waveforms of force and moment signals with a mutual phase shift of 90°. The common load frequency for the entire test was 0.0417 Hz. Other authors used a similar path to validate their estimates [7,9,10,11,12,13,14,15,16].

For the NV path, both channels had sinusoidal waveforms. The loading frequency of the torsion channel was five times faster than that of the tension compression channel. For the digital image correlation (DIC) measurement, which was carried out for the first 49 cycles, frequencies were 0.004 Hz for the torque channel, and 0.02 Hz for the force channel. Other authors used this path to validate other approximation methods [7,8,9,11,14,15].

Path Circle consisted of two sinusoidal waveforms of axial force and torque with a phase shift of 90°. The loading frequency was 0.1 Hz. In contrast to paths Square and NV, which appeared in several publications by other authors, path Circle has so far only been presented in [7,12,16].

Path 7 represents loading by a constant torque in one channel and a sinusoidal waveform of tension–compression in the other. The loading frequency when measuring the strains was 0.1 Hz. Path 7 was used for validation in [7,8] but only in the form of FEA simulations.

Loading frequencies were set according to the ability of the testing machine used to maintain the loading paths on each channel without distortions. The sampling frequency of the DIC cameras was also taken into account, as the loading frequencies had to be 20 times smaller than the sampling frequency of the used cameras to avoid aliasing.

The testing machine used for the experiments was INOVA FU 250 (distributed by Inova Praha s.r.o.), multiaxial tension–compression and torsion load frame with hydraulic actuator for dynamic loading. The maximal value of the axial force channel of the machine is 250 kN, and the maximal moment is 2000 Nm.

The Dantec Dynamics 3D Q-450 high-speed image correlation system was used for the DIC measurement. The system consists of MKII-NanoSense cameras with a CCD sensor with resolution of 1024 × 1280 pixels and Istra 4-D software (version 4.4.3.414). The software was used for calibration, measurement, displacement evaluation, and displacement export.

For calculating axial and shear strains on the basis of exported displacements from Istra 4D, a program was written in MATLAB language. The reason for processing the data outside the DIC system was the possibility of applying a higher level of automation and more control over displacement smoothing.

## 3. Finite Element Analyses

FE analyses with purely elastic and elastic–plastic material data were carried out in Abaqus v6.14-5. The elastic material model aimed to achieve notch tip stress histories to use them as inputs for the approximations. Analyses with elastic–plastic material data were performed to verify the correspondence of the material data and the experimental results.

**Figure 5:**
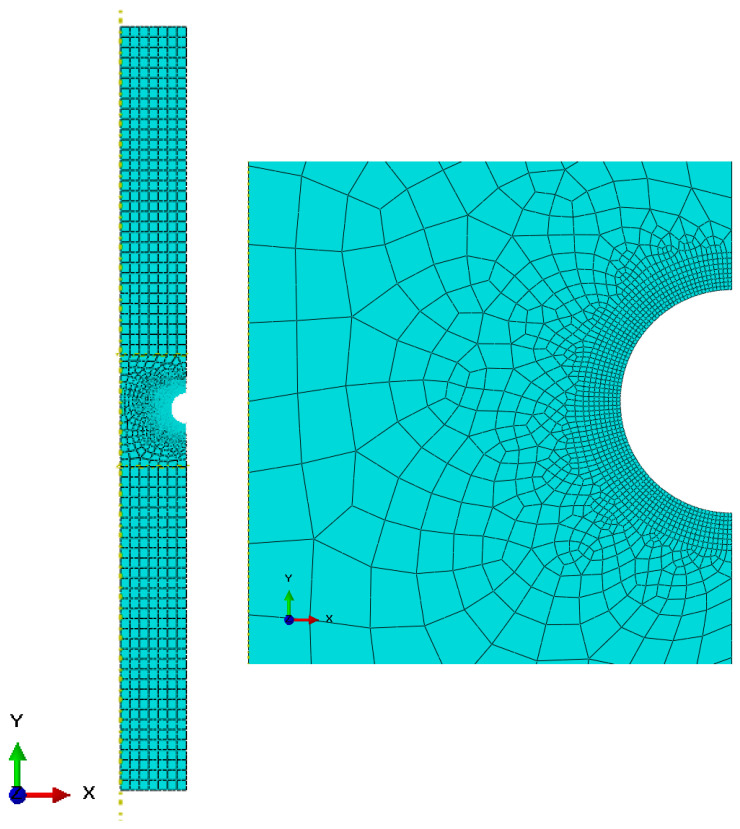
Axisymmetric model of U-notched specimen in Abaqus.

Specimens were modeled as axisymmetric. The same mesh was used for both elastic and elastic–plastic analyses. The final mesh size of the quadratic axisymmetric stress elements CGAX8R (8-node biquadratic, reduced integration) was 0.1 mm (Figure 5). Attempts to further decrease the element size did not affect the results by more than by 0.007%. All elements passed the mesh quality check without errors and warnings.

For elastic–plastic analyses, combined hardening behavior was chosen with the stabilized data type. The number of backstresses was set to 5. The cyclic stress–strain curve used in the model was calculated on the basis of the Hollomon parameters presented in Section 2.3. Its values are shown in Table 3.

**Figure 6:**
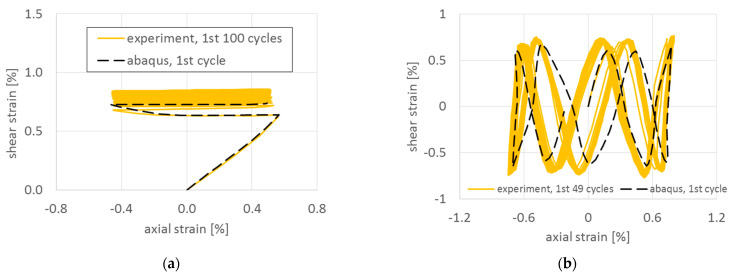
Comparison of experimental data and results of elastic–plastic FEA in Abaqus for paths (**a**) 7 and (**b**) NV.

The coincidence of the responses from the experiments and from FEA (Figure 6) at the initial loading and at the beginning of cyclic loading suggests that the elastic data were valid. There were small differences in cyclic regions that could have been caused by the absence of a ratcheting parameter in the combined hardening model of plasticity in Abaqus.

## 4. Implementation of Approximation Method

The approximation method itself was implemented in MATLAB. The only input for the program was the stress history of the notch tip of the elastic FEA and the material data presented in Section 2.3. The cyclic stress–strain material curve was defined using Hollomon parameters for five sections of plastic strain with a step of 0.02 and the first value corresponding to a cyclic yield strength of 330 MPa. The pseudocurve was established according to the process described in Section 2.1.1.

Parameters of the plasticity model *C_i_* and *γ_i_* were calculated according to Equations (11)–(13).

## 5. Results and Discussion

**Figure 7:**
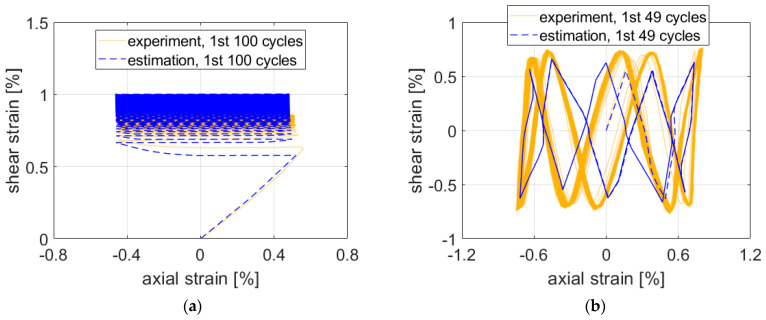
Comparison of experimental data on aluminum alloy 2124-T851 and corresponding estimates for paths (**a**) 7 and (**b**) NV. Parameter μ was set to 0 (OWI model).

**Figure 8:**
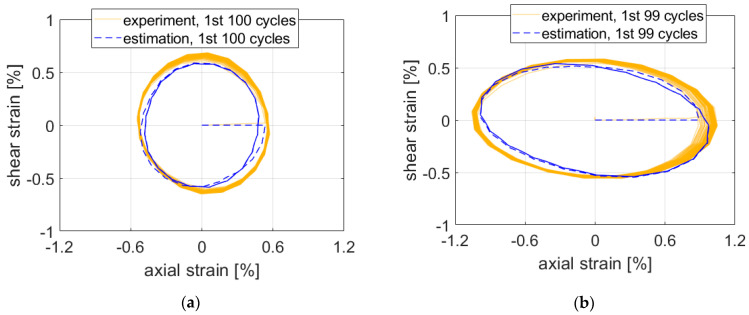
Comparison of experimental data and estimates for path Circle. Ratio of nominal axial and shear stress (**a**) 1 and (**b**) 1.73.

**Figure 9:**
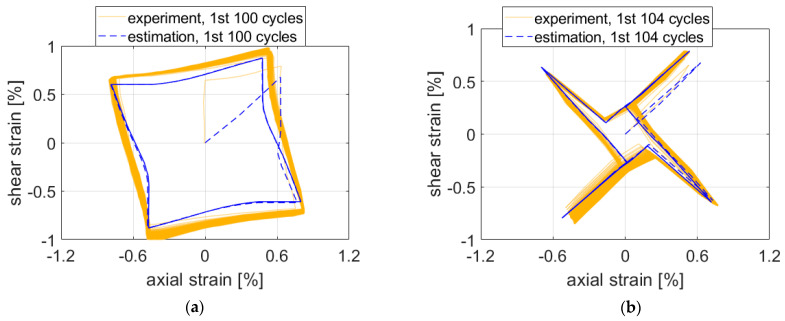
Comparison of experimental data and estimates for paths (**a**) Square and (**b**) X.

First, all results depicted in Figure 7, Figure 8 and Figure 9 are presented for the OWI model (μ=0). The experimental load path patterns shown in ochre correspond to the loading paths depicted in Figure 4, but unlike the controlled forces and moment shown there, the strain counterparts showed ratcheting and subsequent stabilization behavior.

The estimate for Path 7 was the least precise output from the evaluated test cases. Constant mean stress and ratcheting caused by the mean stress are the most probable explanations.

For path NV, the estimate followed the experimental results quite closely (Figure 7b) if the complexity of the path is taken into account. Maximal strain components were underestimated by approx. 0.1–0.2% of strain.

The estimates for path Circle in Figure 8 were slightly nonconservative. Precision seemed to be roughly the same for both stress ratios of nominal to shear stresses.

Similarly to paths Circle and NV, the maximal experimental strain values were not reached by the estimate in the case of path Square (Figure 9a). The approximate solution resulted in greater differences between the elastic and elastic–plastic regions. This response might be caused by the isotropic hardening rule missing in the plasticity model. Adjustments to isotropic hardening parameters might improve the accuracy of the estimation.

However, in the case of path X (Figure 9b), the maximal experimental strain values were exceeded in the first three quadrants. The visible stabilization in the experimental data did not appear in the estimated results when the ratcheting parameter was set to 0.

**Figure 10:**
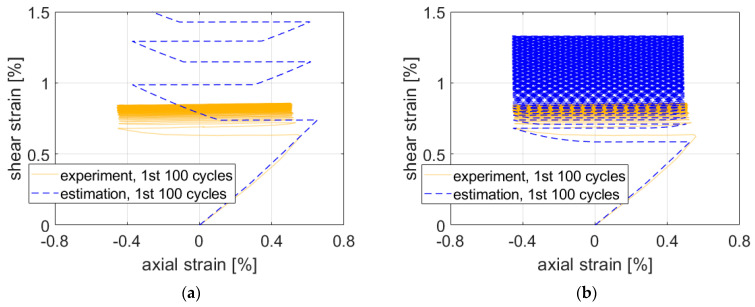
Ratcheting prediction by special cases of the AKO model for Path 7: (**a**) μ=1 (CHAB); (**b**) μ=0.1.

The influence of the ratcheting parameter on the prediction for Path 7 is shown in Figure 10. The value of μ=1 (CHAB) resulted in an excessive accumulation of plastic shear strain from the first cycle (Figure 10a). The value of μ=0.1 (AKO) provided better prediction of the evolution of the shear strain in the first cycle, but prediction after saturation of the response was worse than that of OWI (compare Figure 7a and Figure 10b). Thus, the best prediction of ratcheting is obtained for the limit value of the ratcheting parameter μ=0, which corresponds to the Ohno–Wang I model [26]. However, the ratcheting response remained exaggerated for the 2124-T851 aluminum alloy. One of the possible solutions is to implement the constitutive model of Chen and Jiao with a multiaxial ratcheting parameter [32]. The mentioned model enables one to predict even plastic shakedown under multiaxial stress state for the case with a nonzero mean stress component.

**Figure 11:**
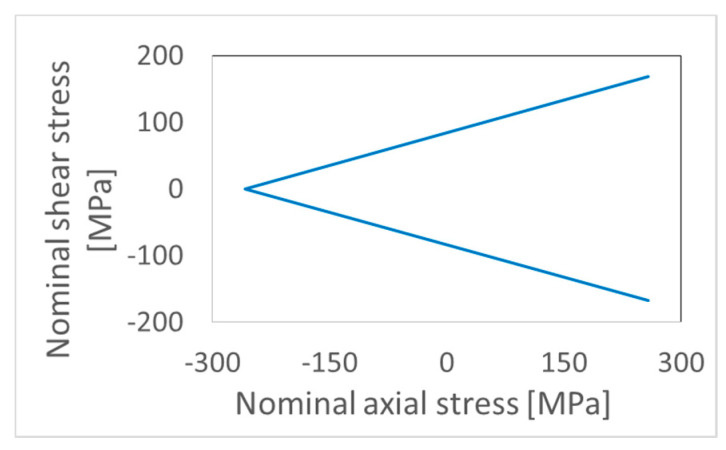
Rotated V-shaped path used for validation of proposed approach on TC21 titanium alloy [14].

In order to validate the proposed approach on other materials, a numerical study was performed. For paths NV and Square, estimates based on data in the literature [14] corresponding to 1070 steel and the TC21 titanium alloy were compared with estimates by Tao et al. [14]. These authors used the results of Barkey in the case of 1070 steel [7], but tests on the TC21 titanium alloy were their own. They were carried out on hollow samples with a perpendicularly drilled hole of 3 mm diameter. The strain records were obtained from 3 strain gages placed into the immediate vicinity of the hole with length and width of 0.5 mm each. In the case of the TC21 titanium alloy, the comparison was also carried out for a rotated V-shaped loading path, see Figure 11. Estimates by Tao et al. were chosen on the basis of their reported quality and because the necessary input data for carrying out own estimates were complete.

Table 4 shows nominal stresses and stress concentration factors used to identify pseudonotch stress history. The components of the pseudonotch stress tensor were calculated as $\sigma_{22}^{e}=K_{t22}S_{22},\;\sigma_{23}^{e}=K_{t23}S_{23},\;\sigma_{33}^{e}=K_{t33}S_{22}$. Remaining components were zeroes due to the plain stress condition.

The material data are those presented in [14] and are shown in Table 5. Plasticity model parameters *C_i_* and *γ_i_* were obtained on the basis of data with the function *calc_C_gamma* (Appendix A). The only difference from the source was the cyclic yield strength for 1070 steel, which was calculated as $\sqrt{3}$ * cyclic yield strength in shear (165 MPa) presented for the same material in [33], and resulted in 286 MPa. This value led to better results than those of the initial yield strength of 242 MPa used for the Garud’s model of plasticity in [14]. The original plots from [14] were recreated using an online tool [34].

**Figure 12:**
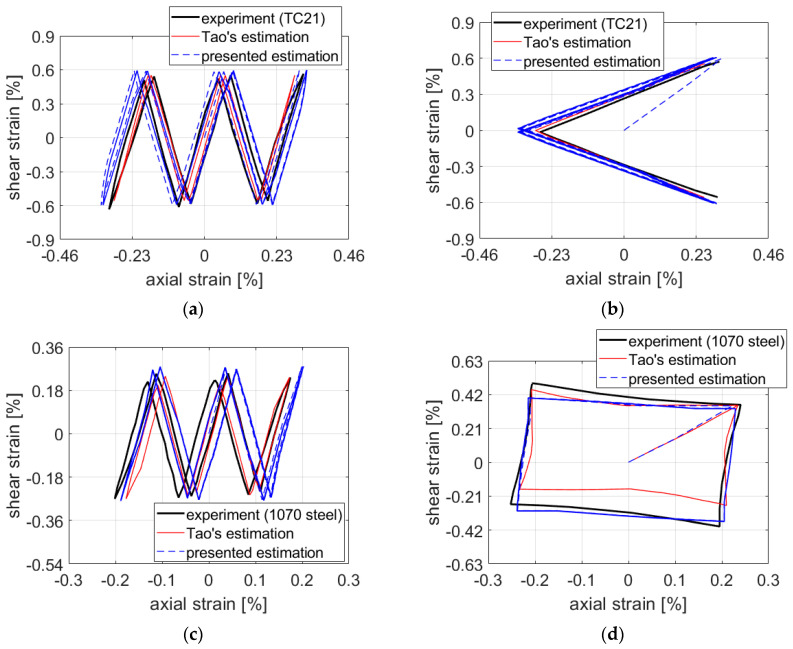
Comparison of experimental data and estimates for (**a**,**b**) TC21 titanium alloy; (**c**,**d**) 1070 steel.

Results of the estimation models are depicted in Figure 12. To quantify the difference between the estimates and experimental results, Tao et al. used the relative errors of strain ranges of individual components. Relative errors *RE* were calculated as follows:

(14) $$RE=\frac{\left(Calculated\;strain\;range-Measured\;strain\;range\right)}{Measured\;strain\;range}.$$

Results using this relative error can be found in Table 6. This metrics for comparing the prediction quality could be discussed (see, e.g., the relative disproportions in the load path shape in Figure 12d, which are not reflected so clearly in Table 6), and other metrics could be proposed. However, its use here allows for us the quantified comparison to the results achieved by Tao et al. in [14]. Figure 12a,c show results corresponding to 1070 steel and TC21 titanium alloy for path NV. Comparing the estimates with the ones presented by Tao et al. using the relative error of strain ranges, results were better for all components (Table 6) except the shear strain in case of 1070 steel that, despite being larger in absolute values, was conservative.

In the case of rotated V-path, the proposed method overestimated both axial and shear strains. A slight nonproportional hardening of TC21 titanium alloy reported in [14] was not taken into account in the calculation, which could have affected the results. We aim to focus on the incorporation of a nonproportional parameter into constitutive equations in a future work.

The estimate for path Square of proposed method provides lesser relative error in strain ranges (Table 6) and more closely followed the experimental data (Figure 12d).

## 6. Conclusions

In the present article, the new combination of the pseudostress approximate method with the Abdel–Karim–Ohno plasticity model was introduced. The combination allows for analysts to estimate elastic–plastic notch tip stresses and strains without performing elastic–plastic FE analysis because only elastic stress history and material data are needed as input. The prediction of ratcheting is not precise; however, it is possible to adjust the amount of ratcheting for different materials by changing the ratcheting parameter *μ*.

The basic parameters for the approximation model can be obtained from standard material data, i.e., from the cyclic stress–strain curve.

The method was tested on previously unpublished experimental data obtained on notched samples manufactured from the 2124-T851 aluminum alloy. Strain components at the notch tip were measured by DIC. The data represent valuable acquisition because only few experimental data suitable for the verification of notch strain components have been presented. None such documented experiment has been carried out on an aluminum alloy.

The method was also tested on data from the literature [14], which included frequently used data on 1070 steel and new experimental data on TC21 titanium alloy.

The estimates agreed well with all tested experimental results and provided competitive quality compared with estimates by other authors.

The common problem of elastic–plastic stress–strain approximation methods is the insufficient description of the methods. This work attempted to fill in the missing details and clarify vague parts. The code of the approximation method in the MATLAB programming language is provided in Appendix A.

## Figures and Tables

**Table 1:** Materials used in other papers for the validation of the methods for calculating the elastic–plastic stress and strain.

Author	Material	Used Data
Barkey [7]	1070 steel	[7]
Koettgen et al. [8]	steel	only FEA
Langlais [9]	1070 steel	[7]
Firat [10]	1070 steel	[7]
Ince et al. [11]	1070 steel	[7]
Ye et al. [12]	S460N steel	[12]
Li et al. [13]	1070 steel, S460N steel	[7,12]
Tao et al. [14]	TC21 titanium alloy, 1070 steel	[7,14]
Li et al. [15]	GH4169 superalloy	only FEA
Kraft [16]	steel	only FEA

**Table 2:** Material parameters of plasticity model obtained for 2124-T851 aluminum alloy.

Real		Pseudo	
Parameter	Value	Parameter	Value
$C_{1}$ (MPa)	16,401	$C_{1}$ (MPa)	51,760
$\gamma_{1}$ (-)	500	$\gamma_{1}$ (-)	500
$C_{2}$ (MPa)	4561	$C_{2}$ (MPa)	7205
$\gamma_{2}\;$(-)	250	$\gamma_{2}\;$(-)	250
$C_{3}$ (MPa)	1948	$C_{3}$ (MPa)	3908
$\gamma_{3}\;$(-)	166.7	$\gamma_{3}\;$(-)	166.7
$C_{4}$ (MPa)	1097	$C_{4}$ (MPa)	2637
$\gamma_{4}$ (-)	125	$\gamma_{4}$ (-)	125
$C_{5}$ (MPa)	4216	$C_{5}$ (MPa)	27,589
$\gamma_{5}\;$(-)	100	$\gamma_{5}\;$(-)	100

**Table 3:** Cyclic stress–strain curve used for elastic–plastic simulation.

Stress (MPa)	Plastic Strain (-)
330	0.000
371.56	0.002
395.19	0.004
409.72	0.006
420.35	0.008
428.78	0.010
435.79	0.012
441.81	0.014
447.09	0.016
451.81	0.018

**Table 4:** Nominal stresses and stress concentration factors used for estimates on experiments concerning 1070 steel and TC21 titanium alloy [14].

Material	Stress Concentration Factors			Loading Path	Nominal Axial Stress *S_22_* (MPa)	Nominal Shear Stress *S_23_* (MPa)
	Tension *K_t22_*	Torsion *K_t23_*	Transverse *K_t33_*			
1070 steel [7]	1.31	1.53	0.27	NV	258	168
				square	296	193
TC21 titanium alloy [14]	1.45	1.17	0.3	NV	299	173
				rotated V	299	173

**Table 5:** Material data on 1070 steel and TC21 titanium alloy used for estimates.

Material	Young’s Modulus	Poisson’s Ratio	Ramberg–Osgood Parameters		Cyclic Yield Strength	Ratcheting Parameter
	(GPa)	(-)	*K* (MPa)	*n* (-)	(MPa)	*μ_i_* (-)
1070 steel [7]	210	0.3	1736	0.199	286	0.3
TC21 titanium alloy [14]	121	0.3	1558	0.093	400	0.1

**Table 6:** Relative errors between measured and calculated strain ranges.

Material	Path	Strain Component	*RE* of Proposed Model (%)	*RE* of Estimate in [14] (%)
1070 steel	NV	Axial	4.26	−5.29
		Shear	7.28	−2.85
	Square	Axial	−4.74	−4.98
		Shear	−13.36	−14.71
TC21 titanium alloy	NV	Axial	5.91	−6.76
		Shear	−0.35	−5.64
	Rotated V	Axial	13.53	−3.83
		Shear	8.20	−3.32

## Data Availability

All data are contained in the present article.

## Appendix A. Implementation of Approximate Method in MATLAB

%% %%%%%%%%%%%%%%%%%%%%%%% GLOBAL VARIABLES

global yieldStrength

%% %%%%%%%%%%%%%%%%%%%% MATERIAL DATA

E = 73100; ny = 0.33; G = E/2/(1 + ny); yieldStrength = 330;

K = 646; % MPa, Hollomon parameters for RO curve

nRO = 0.089;

%% %%%%%%%%%%%%%%%%%%%%%%%%%%%% MATERIAL CURVES

[ePlRC, sRC, ePlPC, sPC] = material_curves(E, K, nRO);

[KPC, nPC] = ramberg_osgood_coefficients(ePlPC, sPC);

%%%%%%%%%%%%%%% PLASTICITY MODEL PARAMETERS – C AND GAMMA

[gPC, cPC] = calc_C_gamma(ePlPC,sPC,KPC,nPC);

[gRC, cRC] = calc_C_gamma(ePlRC,sRC,K, nRO);

%% %%%%%%%%%%%%%%%%%%%%% LOADING PATH

path_name = ‘square’; %‘7’, ‘circle’, ‘NV’, ‘square’, ‘circle_1p73’, ‘X’

define_notch_stress_imputs;

increase_number_of_load_cycles;

%% %%%%%%%%%%%%%%%%%% READ EXPERIMENTAL DATA FOR COMPARISON

load(strcat(pwd,‘/experiments_for_comparison/’,test_spcm,‘_notch_strains_and_info.mat’));

%% %%%%%%%%%%%%%%%%%% PREALOCATING VARIABLES

incPerPlR = 5; prealocate_variables; % incPerPlR per plastic range

%% %%%%%%%%%%%%%%%%%%%%%%%%% THE ESTIMATION PROCEDURE

ii = 1; % index of estimation points, all matrices at 1 consist of zeros.

for i = 2:1:(length(sigYyFI))

  % i … index through loading steps

  SPsStart = SPs; % deviatoric pseudostress at the beginning of the step

          alfaPsI = alfaPs; % I as initial

          ii = ii+1;

            %% find the start of the plasticization

          sigPs = [0        0                    0;

              0        sigYyFI(i                    sigYzFI(i);

              0        sigYzFI(i)        sigZzFI(i);];

SPs        = sigPs      - trace(sigPs)/3*eye(3);      % pseudostress deviator

          % find start of the plasticization

          tol = 1;   %(MPa), tolerance

          beginAt = 0.01;

          for i2 = beginAt:beginAt/10:1

            SInt = SPsStart+(SPs-SPsStart) * i2;

sEq = calc_equiv_stress(SInt-alfaPsI);

if (sEq - yieldStrength) < tol && (sEq - yieldStrength) > 0

elastic_part_instep = 1; break;

elseif (sEq - yieldStrength) > 0

                      % no purely elastic loading during the step

elastic_part_instep = 0; break;

end

end

% check if elastic loading/unloading has occurred

if i2 ~= beginAt

%% PURELY ELASTIC region solution

dSigYPs =(sigYyFI(i)-sigYyFI(i-1))*i2;dSigZPs =(sigZzFI(i)-sigZzFI(i-1))*i2;

dSigYzPs = (sigYzFI(i)-sigYzFI(i-1))*i2;

elastic_region_solution; % >> function

                epsXxPl(ii) = epsXxPl(ii-1);epsYyPl(ii) = epsYyPl(ii-1);

epsZzPl(ii) = epsZzPl(ii-1);epsYzPl(ii) = epsYzPl(ii-1);

else

% no purely elastic loading during the step

sigYPs( ii)=sigYyFI(i-1);sigZPs( ii)=sigZzFI(i-1); sigYzPs(ii)=sigYzFI(i-1);

ii= ii-1; % in order to not skip index when elastic variables weren’t calculated

end

%% plastic region solution (elastic+plastic stresses and strains)

if i2 < 1

% plasticization has occurred

                % pseudonotch stresses increments till the end of current step

dSigYPs =(sigYyFI(i) - sigYPs( ii))/incPerPlR;

dSigZPs =(sigZzFI(i) - sigZPs( ii))/incPerPlR;

dSigYzPs = (sigYzFI(i) - sigYzPs(ii))/incPerPlR;

for j = 1:1:incPerPlR

%% cycle trough increments of plastic range

                         ii = ii + 1; % index of calculated variables – stresses and strains

sigYPs(ii) = sigYPs(ii-1)+dSigYPs;sigZPs(ii) = sigZPs(ii-1)+dSigZPs;

sigYzPs(ii) = sigYzPs(ii-1)+dSigYzPs;% pseudostress

                         sigPs = [0   0          0;

0 sigYPs(ii) sigYzPs(ii);

0 sigYzPs(ii) sigZPs(ii)];

                         SPs = sigPs- trace(sigPs)/3*eye(3);% deviator of pseudostress

                         [dp,theta] = calc_dp_AKO(gPC,cPC,SPs,alfa_partPs);

  [alfaPs, alfa_partPs, dEpsPlPs] = …

  calc_alfa_and_dEpsPl(dp,gPC,cPC,alfa_partPs,SPs, theta);

  dEpsPl = dEpsPlPs;

  [alfa, alfa_part, SReal] = …

  calc_alfa_and_Snp1(dp,gRC,cRC,alfa_part,dEpsPl);

  %% non-deviatoric real stress components

  sigRealH = -SReal(1,1);%% plain stress condition

  sigReal = SReal + sigRealH*eye(3);

                         sigY(ii) = sigReal(2,2);sigZ(ii) = sigReal(3,3);sigYz(ii) = sigReal(2,3);

  dSigY = sigY(ii) - sigY(ii-1);dSigZ = sigZ(ii) - sigZ(ii-1);

                         dSigYz = sigYz(ii) - sigYz(ii-1);

  %% storing calculated strains

  % plastic strain increments - tensorial shear strain form!

                         x(1) = dEpsPl(1,1); x(2) = dEpsPl(2,2); x(3) = dEpsPl(3,3); x(4) = dEpsPl(2,3);

  % elastic strain

  dEpsXxE = 1/E * (-ny) * (dSigY + dSigZ);

  dEpsYyE = 1/E * (dSigY + (-ny) * dSigZ);

  dEpsZzE = 1/E * (dSigZ + (-ny) * dSigY);dGammaYzE = 1/G* dSigYz;

  dEpsYzE = dGammaYzE/ 2;

                         epsXxE(ii) = epsXxE(ii-1) + dEpsXxE; epsYyE(ii) = epsYyE(ii-1) + dEpsYyE;

  epsZzE(ii) = epsZzE(ii-1) + dEpsZzE;epsYzE(ii) = epsYzE(ii-1) + dEpsYzE;

  % plastic strain

  epsXxPl(ii) = epsXxPl(ii-1) + x(1); epsYyPl(ii) = epsYyPl(ii-1) + x(2);

  epsZzPl(ii) = epsZzPl(ii-1) + x(3);epsYzPl(ii) = epsYzPl(ii-1) + x(4);

  % total strain (elastic total new + plastic total)

  epsXx(ii) = epsXxE(ii) + epsXxPl(ii); epsYy(ii) = epsYyE(ii) + epsYyPl(ii);

  epsZz(ii) = epsZzE(ii) + epsZzPl(ii);epsYz(ii) = epsYzE(ii) + epsYzPl(ii);

end %j = 1:1:(incPerPlR)

end

end

graphs; % function for plotting results

%% %%%%%%%%%%%%%%%%%% FUNCTIONS: %% %%%%%%%%%%%%%%%

function [ePlRC, sRC, ePlPC, sPC] = material_curves(E, K, nRO)

% RC … real curve (cyclic stress strain curve); Pl … plastic

% PC … pseudocurve;e … epsilon, s … sigma

%% REAL CURVE

% 4 intervals for gamma and C and 1 corresponding to the yield

% strength - added later -> 5 intervals == 5 backstress parts

ePlRC = [0.002 0.004 0.006 0.008 0.01]′;

n = size(ePlRC,1); %number of discrete points on the curve

for i = 1:n

  sRC(i,1) = K*ePlRC(i)^nRO;

end

%% PSEUDOCURVE

ePlPC= ePlRC;

for i = 1:n

%sig_e = sig *eps_tot*E

sPC(i,1) = sqrt( sRC(i)* (ePlRC(i)+sRC(i)/E) *E );

end

end %% %%%%%%%%%%%%%%%%%%%%%%%%%%%%%% END OF FUNCTION

function [K, n] = ramberg_osgood_coefficients(eps, sig)

f = fit(eps,sig,‘power1’);

regCs = coeffvalues(f);

K = regCs(1); %RO with Hollomon parameters: eps = sig/E + (sig/K)^(1/n)

n = regCs(2);

end %% %%%%%%%%%%%%%%%%%%%%%%%%%%%%%% END OF FUNCTION

function [gamma, cMatrix] = calc_C_gamma(epsPl,stress,K,n)

global yieldStrength

slope = zeros(size(epsPl,1),1);gamma = zeros(size(epsPl,1)-1,1);

cMatrix = zeros(size(epsPl,1)-1,1);epsPlAtYS = (yieldStrength/K)^(1/n); nn = size(epsPl,1);

for i = 1:nn

if i == 1

slope(1) = (stress(1)-yieldStrength)/(epsPl(i)-epsPlAtYS);

else

slope(i) = (stress(i)-stress(i-1))/(epsPl(i)-epsPl(i-1));

end

end

slope(nn+1) = 0;

for i = 1:nn

gamma(i) = 1/epsPl(i);

cMatrix(i) = slope(i) - slope(i+1);

end

end %% %%%%%%%%%%%%%%%%%%%%%%%%%%%%%% END OF FUNCTION

%% %%%%%%%%%%%%% PROCEDURE define_notch_stress_imputs.m

if strcmp(path_name,‘square’)

% Path Square,F = 66 kN, M = 329.133 Nm, D 26 mm

test_spcm = ‘u3’;

num_cycles_text = ‘1st 100 cycles’;

sigYyFI = [0112 109 109 -3-116-120-120-7 112 ];

sigZzFI = [0427 428 428 0 -429-427-4271427];

sigYzFI = [0-291 1 292 292 292 1 -291-291 -291];

elseif strcmp(path_name,‘NV’)

% Path NV;F = 66 kN, M = 329.133 Nm, D 26 mm

test_spcm = ‘u4’;

num_cycles_text = ‘1st 49 cycles’;

% coarse input - multilinear

sigYyFI = [0366591105 112 104 906333-4-37 -71 -95 …

  -113 -117-114-96 -73 -41 -8];

sigZzFI = [0132 252 346 407 428 408 346 253 133 1 -131-250 …

  -345 -405-427-405-345-249-1303];

sigYzFI = [0-2920 292 0 -2920 292 0 -2920 …

  2910 -2910 291 0 -2910 291 0];

elseif strcmp(path_name,‘circle’)

% Path Circle;F = 66 kN, M = 329.133 Nm, D 26 mm

test_spcm = ‘u73’;

num_cycles_text = ‘1st 100 cycles’;

sigYyFI = [0112 106 9167361 -34 -66 -91 -108-114 …

  -108 -92 -67 -35 0 356590105 110];

sigZzFI = [0427 406 345 251 131 -1-133-253-347-408 …

  -429 -408-347-253-133-1131 251 345 406 427];

sigYzFI = [00 90172 236 277 292 277 236 171 900 -90 -171 …

  -236 -277-291-277-236-171-90 0];

elseif strcmp(path_name,‘circle_1p73’)

          % F = 101 kN, M = 290 Nm, D 26 mm

          sigYyFI = [0170 170 162 138 101 531 -53 -101-140-165-174-165-140-102-53 0 53100 137 161 169];

          sigZzFI = [0651 651 619 527 382 200 -2-204-386-531-624-656-624-531-386-204-2200 382 526 619 651];

          sigYzFI = [00 0 80151 208 245 257 244 208 151 790 -79 -151-208-244-257-245-208-151-80 0];

elseif strcmp(path_name,‘7’)

% Path “7”; F = 66 kN, M = 329.133 Nm, D 26 mm

test_spcm = ‘f9’;

num_cycles_text = ‘1st 100 cycles’;

sigYyFI = [080.8 -78.280.7];% tangential direction

sigZzFI = [0373.3-371.7 372.9]; % axial direction,

sigYzFI = [0 -271.4-270.5-271.5]; % FI as for incrementation

elseif strcmp(path_name,‘X’)

% F = 66 kN, M = 329.133 Nm, D 26 mm

sigYyFI = [0112-4109-7-120-10-123-14];

sigZzFI = [0427 04281-428 1 -428 1];

sigYzFI = [0 -292 12920 292 1 -291 0];

end

%% %%%%%%%%%%%%%%%%%%%%%%%%%%%%%% END OF PROCEDURE

%% %%%%%%%%%%%%% PROCEDURE prealocate_variables.m

n = size(gPC,1);% number of backstress parts

alfa = zeros(3,3);% total backstress

alfa_part = zeros(3,3,n); % backstress parts

alfaPs = zeros(3,3);% total pseudobackstress

alfa_partPs = zeros(3,3,n); % parts of the pseudobackstress

alfa_partPsI = zeros(3,3,n); % parts of the pseudobackstress at the beginning of the step

theta = ones(1,n);

SPs = zeros(3,3);SPsStart = zeros(3,3);SReal = zeros(3,3);

dEpsPl = zeros(3,3);dEpsPlPs = zeros(3,3); dp = 0;

if sigYyFI(length(sigYyFI)) == 0

% m = number of points in elastic range + number of points in plastic

%range + one initial zero state

m = length(sigYyFI)-1 + incPerPlR*(length(sigYyFI)-2) + 1;

else

m = length(sigYyFI)-1 + incPerPlR*(length(sigYyFI)-1) + 1;

end

epsXx(1) = 0; epsYy(1) = 0; epsZz(1) = 0; epsYz(1) = 0;

epsXxE(1) = 0;epsYyE(1) = 0;epsZzE(1) = 0;epsYzE(1) = 0;

epsXxPl(1) = 0; epsYyPl(1) = 0; epsZzPl(1) = 0; epsYzPl(1) = 0;

sigY = zeros(m, 1); sigZ = zeros(m, 1); sigYz = zeros(m, 1);

sigYPs(1) = 0; sigYPs(2) = 0; sigZPs = zeros(m, 1); sigYzPs = zeros(m, 1);

%% %%%%%%%%%%%%%%%%%%%%%%%%%%%%%% END OF PROCEDURE

function equivalent_stress = calc_equiv_stress(A)

aMatrix = A.*A;

equivalent_stress = sqrt(3/2* sum(aMatrix(:)) );

end %% %%%%%%%%%%%%%%%%%%%%%%%%%%%%%% END OF FUNCTION

%% %%%%%%%%%%%%% PROCEDURE elastic_region_solution.m

sigYPs(ii)= sigYPs(ii-1) + dSigYPs;sigZPs(ii)= sigZPs(ii-1) + dSigZPs;

sigYzPs(ii) = sigYzPs(ii-1)+ dSigYzPs;sigY(ii)= sigY(ii-1) + dSigYPs;

% in elastic regime increments of real and pseudostresses are the same

sigZ(ii)= sigZ(ii-1) + dSigZPs;sigYz(ii) = sigYz(ii-1)+ dSigYzPs;

sigReal = [0 0 0;

  0 sigY(ii)sigYz(ii);

  0 sigYz(ii) sigZ(ii)];

SReal = sigReal - trace(sigReal)/3*eye(3);

dEpsXxE = 1/E * (-ny) * (dSigYPs + dSigZPs);dEpsYyE = 1/E * (dSigYPs + (-ny) * dSigZPs);

dEpsZzE = 1/E * (dSigZPs + (-ny) * dSigYPs);dGammaYzE = 1/G *dSigYzPs;

dEpsYzE = dGammaYzE/ 2;

epsXxE(ii) = epsXxE(ii-1) + dEpsXxE;epsYyE(ii) = epsYyE(ii-1) + dEpsYyE;

epsZzE(ii) = epsZzE(ii-1) + dEpsZzE;epsYzE(ii) = epsYzE(ii-1) + dEpsYzE;

epsXx(ii) = epsXx(ii-1) + dEpsXxE;epsYy(ii) = epsYy(ii-1) + dEpsYyE;

epsZz(ii) = epsZz(ii-1) + dEpsZzE;epsYz(ii) = epsYz(ii-1) + dEpsYzE;

%% %%%%%%%%%%%%%%%%%%%%%%%%%%%%%% END OF PROCEDURE

function [dp,theta] = calc_dp_AKO(gamma,cMatrix,S,alfa_part)

global yieldStrength

n = size(gamma,1);theta = ones(1,n);% vectors prealocation

mu1 = 0; % ratcheting parameter; 0 … OWI; 0.1 … AKO; 1 … CHAB

dpkm1 = 0;dpkm2 = 0;% variables to check convergence

for k =1:100

aMatrix = zeros(3,3); % a supportive variable for the calculation

aNum = 0;

for i2 = 1:n

  aMatrix = aMatrix + theta(i2)*alfa_part(:,:,i2);

  aNum = aNum + cMatrix(i2)*theta(i2);

end

aNum2 = calc_equiv_stress(S-aMatrix);

dp = (aNum2 - yieldStrength)/aNum; % dp from the first iteration

% The Aitken’s delta^2 process to shorten the convergence -----

if mod(k,3) == 0

con = dp-(dp-dpkm1)*(dp-dpkm1)/(dp-2*dpkm1+dpkm2);

if con > 0

  dp = con;

end

end % ---------------------------------------------------------

SminusA = yieldStrength/(yieldStrength+aNum*dp)*(S-aMatrix);

dEpsPl = 3/2*dp*SminusA/yieldStrength;

if abs(1-dpkm1/dp) < 10^(-4)

break; % solution found

end

for i2 = 1:n

aMatrix = zeros(3,3);alnp1_st(:,:,i2) = alfa_part(:,:,i2)+2/3*cMatrix(i2)*dEpsPl;

alnp1_dash(i2) = calc_equiv_stress(alnp1_st(:,:,i2));

                             mu(i2) = mu1;c(i2) = 1/(1+mu(i2)*gamma(i2)*dp);

                             alnp1_hash(:,:,i2) = c(i2)*(alnp1_st(:,:,i2));

                             fnp1_hash = calc_equiv_stress(alnp1_hash(:,:,i2))^2-(cMatrix(i2)/gamma(i2))^2;

theta(i2)=c(i2)+heaviside(fnp1_hash)*…

  (cMatrix(i2)/gamma(i2)/alnp1_dash(i2)-c(i2));

              end

  dpkm2 = dpkm1;dpkm1 = dp;

  if k == 100

  error(‘Error: number of iterations has exceeded the allowed value’);

  k

  end

end

end %% %%%%%%%%%%%%%%%%%%%%%%%%%%%%%% END OF FUNCTION

function [alfa, alfa_part, dEpsPl] = …

calc_alfa_and_dEpsPl(dp,gamma,cMatrix,alfa_part,S,theta)

global yieldStrength

m2 = size(gamma,1); % number of backstress parts

aMatrix = zeros(3,3);alfa = zeros(3,3);aNum = 0;

for i = 1:m2

aMatrix =aMatrix + theta(i)*alfa_part(:,:,i);aNum = aNum + cMatrix(i)*theta(i);

end

% plastic strain tensor

SminusA = yieldStrength/(yieldStrength+aNum*dp)*(S-aMatrix);

dEpsPl = 3/2*dp*SminusA/yieldStrength;% calculate backstress parts (for the next iteration)

for i = 1:m2

alfa_part(:,:,i) = (alfa_part(:,:,i)+2/3*cMatrix(i)*dEpsPl)*theta(i);

alfa = alfa + alfa_part(:,:,i);

end

end %% %%%%%%%%%%%%%%%%%%%%%%%%%%%%%% END OF FUNCTION

function [alfa, alfa_part, Snp1] = …

calc_alfa_and_Snp1(dp,gamma,cMatrix,alfa_part,dEpsPl)

global yieldStrength

m2 = size(gamma,1); % number of backstress parts

alfa = zeros(3,3);

% calculate backstress parts

for i = 1:m2

alfa_part(:,:,i) = (alfa_part(:,:,i)+2/3*cMatrix(i)*dEpsPl)/(1+gamma(i)*dp);

alfa = alfa + alfa_part(:,:,i);

end

Snp1 = dEpsPl*yieldStrength*2/3/dp + alfa;

end %% %%%%%%%%%%%%%%%%%%%%%%%%%%%%%% END OF FUNCTION
